# BarleyNet: A Network-Based Functional Omics Analysis Server for Cultivated Barley, *Hordeum vulgare* L.

**DOI:** 10.3389/fpls.2020.00098

**Published:** 2020-02-18

**Authors:** Sungho Lee, Tak Lee, Sunmo Yang, Insuk Lee

**Affiliations:** Department of Biotechnology, College of Life Science and Biotechnology, Yonsei University, Seoul, South Korea

**Keywords:** barley, *Hordeum vulgare* L., gene network, network biology, crop systems genetics

## Abstract

Cultivated barley (*Hordeum vulgare* L.) is one of the most produced cereal crops worldwide after maize, bread wheat, and rice. Barley is an important crop species not only as a food source, but also in plant genetics because it harbors numerous stress response alleles in its genome that can be exploited for crop engineering. However, the functional annotation of its genome is relatively poor compared with other major crops. Moreover, bioinformatics tools for system-wide analyses of omics data from barley are not yet available. We have thus developed BarleyNet, a co-functional network of 26,145 barley genes, along with a web server for network-based predictions (http://www.inetbio.org/barleynet). We demonstrated that BarleyNet's prediction of biological processes is more accurate than that of an existing barley gene network. We implemented three complementary network-based algorithms for prioritizing genes or functional concepts to study genetic components of complex traits such as environmental stress responses: (i) a pathway-centric search for candidate genes of pathways or complex traits; (ii) a gene-centric search to infer novel functional concepts for genes; and (iii) a context-centric search for novel genes associated with stress response. We demonstrated the usefulness of these network analysis tools in the study of stress response using proteomics and transcriptomics data from barley leaves and roots upon drought or heat stresses. These results suggest that BarleyNet will facilitate our understanding of the underlying genetic components of complex traits in barley.

## Introduction

Cultivated barley (*Hordeum vulgare* L.) is one of the first cultivated grains, domesticated about 10,000 years ago in the Near East (Badr et al., 2000). It was ranked the fourth cereal crop in quantity produced after maize, bread wheat, and rice in 2017 (FAOSTAT 2017, http://fao.org/faostat/). Barley mainly serves as a source of fodder for livestock, fermentable material for alcoholic beverages, and is present in various healthy organic foods. In developing countries, it is also still a major source of carbohydrates. Furthermore, barley is a great plant model organism for studying genetic resistance to biotic or abiotic stress, since it can endure a great range of environmental stresses like drought, flood, and cold or fungal infections, either single or combined (Gürel et al., 2016). Therefore, the barley genome is a reservoir of numerous stress response alleles, which are precious subjects for genetic engineering in other crop species. The size of the haploid Barley genome is approximately 5.3 Gbp. It is one of the largest diploid genomes sequenced to date and contains 83,105 putative genetic loci including 39,734 high-confidence ones.

Numerous studies have exploited these agronomically important traits, assisted by various new technologies such as high-throughput sequencing and mass spectrometry-based proteomics. Although they provide important clues about molecular components associated with complex plant traits, individual omics profiles are insufficient to reconstruct a holistic view of functional modules involved in these traits. Moreover, the functional interpretation of omics profile data generally requires the incorporation of other information. Therefore, a systems biology platform that integrates information derived from different data sources could effectively encapsulate the molecular network underlying complex traits. Co-functional gene networks have been applied to integrate the functional information of genes derived from heterogeneous data through a Bayesian statistics framework (Shim et al., 2017). Co-functional networks previously constructed for other major crop species have been successfully used in the genetic dissection of complex plant traits (Lee et al., 2015a; Lee et al., 2017; Lee et al., 2019). Yet, such an effective network-assisted systems genetics platform has not been developed for barley. Therefore, we developed BarleyNet, a co-functional network of barley genes and a companion web server (www.inetbio.org/barleynet/), enabling network-assisted systems genetics analysis for cultivated barley. All information on functional association between barley genes is also readily downloadable through the companion web server. Finally, the three complementary network-based algorithms implemented in the web server facilitate effective use of omics profiles for generating new functional hypotheses.

## Materials and Methods

### Reference Genome

We constructed BarleyNet based on the IBSC_v2 barley genome assembly (https://plants.ensembl.org/Hordeum_vulgare/Info/Annotation/#assembly) presented by the International Barley Sequencing Consortium (Mascher et al., 2017). Among 83,105 putative genetic loci, 39,734 high-confidence loci were selected as a reference gene set for network construction. Supervised learning of co-functional gene pairs requires gold standard (GS) positive and negative gene pairs, which are generally derived from high-quality pathway annotation databases. However, both the quantity and the quality of pathway annotations for barley were not sufficient by the time we launched this project. Thus, we transferred GS-positive barley gene pairs based on sequence homology with those used for modeling *Arabidopsis* (Lee et al., 2015b) and rice (Lee et al., 2015a) gene networks. Consequently, 215,170 and 27,254 GS-positive gene pairs were transferred from rice and *Arabidopsis*, respectively. The final set of GS-positive gene pairs for training BarleyNet was a union of all transferred gene pairs, comprising 234,070 gene pairs among 7,350 barley genes (18.5% of the genome). All other possible pairwise relationships between the 7,350 barley genes were then considered GS-negatives, comprising 26,773,505 gene pairs.

### Benchmarking Co-Functional Barley Gene Pairs

The likelihood of a functional association between two genes is based on the ratio between our belief after seeing the supporting data and our prior belief. Thus, we scored functional association between genes using previously developed log likelihood score (*LLS*) (Lee et al., 2004), shown as the following equation:

$$LLS=\;\ln\left(\frac{P\left(L|S\right)/P(⌐L|S)}{P\left(L\right)/P\left(⌐L\right)}\right)$$

where *P*(*L*|*S*) and *P*(⌐*L|S*) represent the probability of GS-positive and GS-negative gene pairs, respectively, supported by the given data, and P(*L*) and *P*(⌐*L*) represent the expected probability of GS-positive and GS-negative links, respectively.

Gene pairs are sorted by data-intrinsic scores such as the expression correlation coefficient, and then assigned into bins of 1,000 gene pairs. We computed *LLS* for each of the bins and then did a sigmoid regression between means of data-intrinsic scores and *LLS*s. Using the regression function, we calculated *LLS* for every gene pair derived from each data source.

### Integrating Co-Functional Barley Gene Pairs

Functional association between barley genes can be supported by multiple data sources. We may integrate the *LLS* of their functional association by naïve Bayes integration, if there is no correlation between data sources, which is generally not true. In order to handle information correlation between supporting data sources, we previously developed the weighted sum (*WS*) method (Lee et al., 2007), shown as the following equation:

$$WS=Lo+\sum_{i=1}^{n}\frac{L_{i}}{D\times i},\;for\;all\;L\geq T$$

where *L_O_* represents the highest *LLS* of all available supporting data sources, and *L*_i_ represents the remaining *LLS*s with rank index *i*. *D* and *T* are free parameters for the weight factor and *LLS* cutoff to be considered, respectively. These free parameters were selected where the integrated network achieved the best performance based on a precision-recall curve. A total of 25 distinct data sources were finally integrated into BarleyNet (**Supplementary Table 1**).

### Inferring Co-Functional Links From mRNA Co-Expression Patterns (CX)

Functionally associated genes tend to show a similar expression pattern across various biological contexts. Co-functional links between these genes were inferred from diverse sets of expression profiles gathered from the Gene Expression Omnibus (GEO) database (Clough and Barrett, 2016), ArrayExpress (Kolesnikov et al., 2015), and Expression Atlas (Papatheodorou et al., 2018). We assessed a total of 2,385 expression profiles (1,780 by microarray and 650 by RNA-seq) and incorporated 28 datasets comprising 2,047 expression profiles into the final co-expression network. Affymetrix microarray data (Barley genome array, GPL1340) were normalized by MAS5 software. RNA-seq data were downloaded as raw data, quantified using Kallisto (Bray et al., 2016), and normalized as transcripts per million (TPM). The co-expression between two genes across expression profiles was assessed by the Pearson's correlation coefficient (*PCC*) and then benchmarked for functional associations by *LLS*. All the co-expression networks from the 28 expression datasets (**Supplementary Table 2**) were then integrated into a single co-expression network using the weighted sum method described above.

### Inferring Co-Functional Links From Protein Domain Profile Association (DP)

The domain composition of a protein reflects its function. Therefore, the co-functional relationship between proteins can be inferred from the association between their domain composition profiles. We downloaded a list of barley proteins and identified domains in the InterPro database (Mitchell et al., 2018) for each protein from the Ensembl Plants database (Vullo et al., 2017). Then, mutual information scores were computed between domain profiles. We used a weighted mutual information (*WMI*) scheme, which assigns more weight on rarer domains during mutual information computation (Shim and Lee, 2016; Shim and Lee, 2020). We calculated *LLS*s for gene pairs using a regression function between *WMI* and *LLS*.

### Inferring Co-Functional Links From Phylogenetic Profile Associations (PG)

During speciation, genes that operate the same biological processes tend to be inherited together. Therefore, we can infer co-functional gene pairs based on their co-inheritance pattern across a large number of species. Considering that gene inheritance across species can be represented as phylogenetic profiles, these can be used in the identification of co-inherited genes. We first aligned all the 39,734 barley protein sequences against total protein sequences from 1,626 bacterial genomes, 396 eukaryotic genomes, and 122 archaea genomes using BLASTP (Altschul et al., 1990), and then constructed phylogenetic profiles based on –log(E-value) of BLAST hit scores. Previously, we found that domain-specific phylogenetic profile analysis improved inference of co-functional links (Shin and Lee, 2015). Therefore, we calculated mutual information between two phylogenetic profiles for each of the three domains of life, resulting in three networks for profiles with bacterial, eukaryotic, and archaeal genomes. The resulting networks were scored by *LLS* and integrated into one single network for the phylogenetic profile method.

### Inferring Co-Functional Links From Gene Neighborhood (GN)

Prokaryotic genes that operate in the same biological process tend to be located closely in chromosomes, often forming operons. We thus can infer functional associations between barley genes based on the proximity of their orthologs in prokaryotic genomes with two complementary measures: distance-based approach and probability-based approach (Shin et al., 2014; Szklarczyk et al., 2017). Considering 122 archaeal genomes and 1,626 bacterial genomes, the resulting two networks obtained by the different gene neighborhood measures were then scored by *LLS* and integrated into a single co-functional network for the gene neighborhood method.

In addition, we inferred co-functional links between barley genes from ortholog neighborhoods in metagenomes (Kim and Lee, 2017), which provide tremendous amounts of bacterial contigs. We used two distinct metagenomics resources, the Human Microbiome Project (HMP) database (Huttenhower et al., 2012) and the global ocean microbiome database from the TARA Oceans study (Sunagawa et al., 2015). We used DIAMOND, a fast sequence aligner (Buchfink et al., 2014), due to the enormous number of metagenomic contigs. Inferred co-functional links were scored by *LLS* and integrated with those based on neighborhood in fully sequenced prokaryotic genomes into a single network.

### Inferring Co-Functional Links by Transferring Orthologous Gene Pairs From Other Species

Not only individual genes but also pathways are functionally conserved during speciation. Therefore, we may transfer functional information of orthologous gene pairs between species. This conserved co-functional relationship is called *associalog* (Kim et al., 2013). For protein homology mapping between barley and other species, we used InParanoid (Remm et al., 2001), which provides sensitive orthology mapping by taking account of co-orthologs. Associalogs were then transferred from a total of 21 co-functional networks for nine other species: AraNet v2 (Lee et al., 2015b), MaizeNet (Lee et al., 2019), RiceNet v2 (Lee et al., 2015a), HumanNet v2 (Hwang et al., 2018), MouseNet v2 (Kim et al., 2015), DanioNet (Shim et al., 2016), WormNet v3 (Cho et al., 2014), FlyNet (Shin et al., 2015), and YeastNet v3 (Kim et al., 2014).

### Codes and Data Availability

Source codes for network search functions and edge information of BarleyNet are freely available from github (https://github.com/netbiolab/BarleyNet/).

## Results and Discussion

### Construction of Barleynet *via the* Integration of Omics Data From Barley and Many Other Species

**Figure 1:**
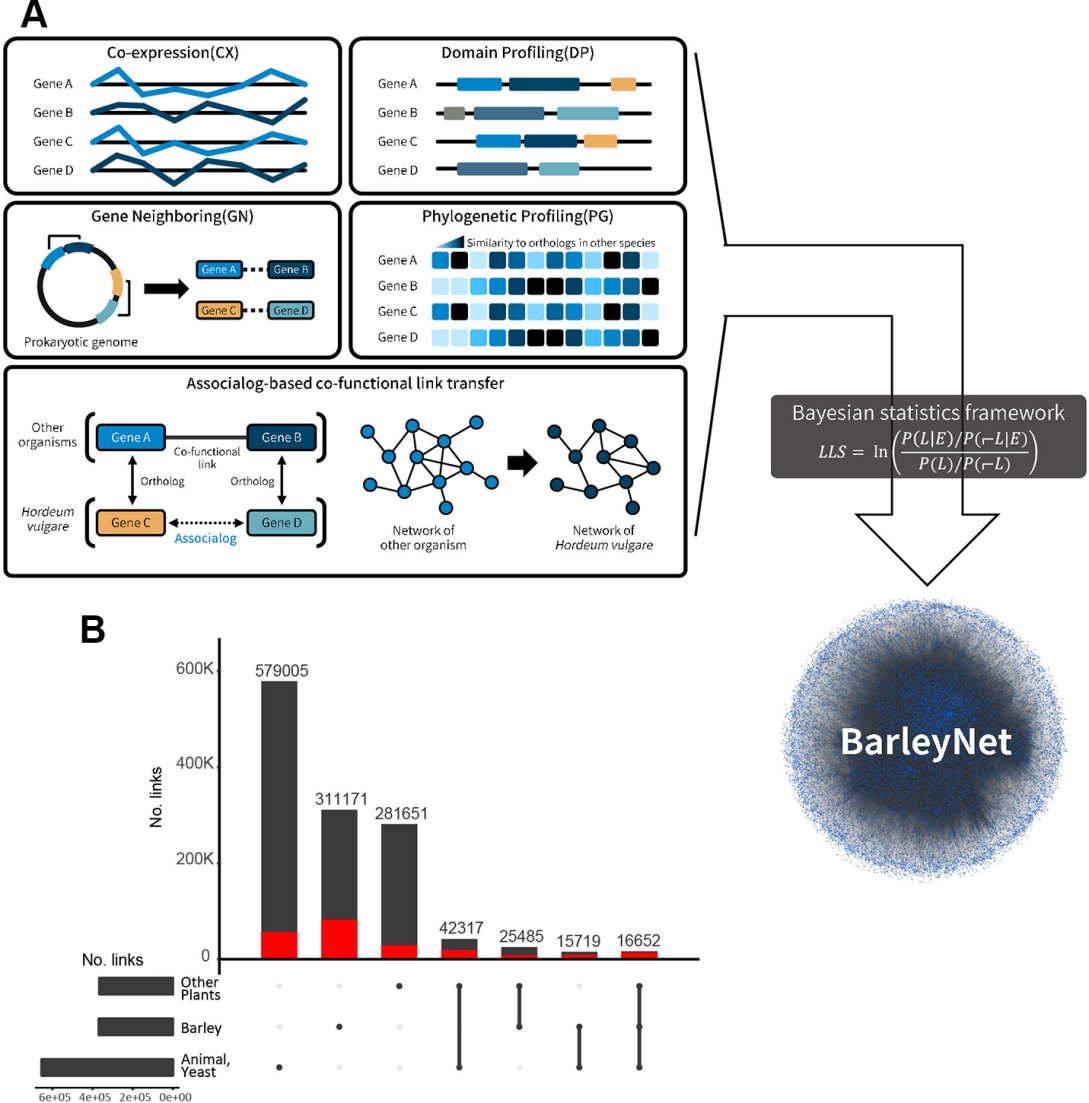
Overview of BarleyNet. **(A)** BarleyNet was constructed by integrating functional associations between barley genes inferred from the co-expression of genes (CX), gene neighborhood (GN), association of protein domain composition profiles (DP), phylogenetic profile association (PG), and those transferred from 21 networks previously constructed for other species based on functional association between orthologous proteins (associalog). **(B)** Summary of BarleyNet edge information with UpSet visualization. Network edges were classified into three groups based on the species of origin of the inferred co-functional association: “barley,” “other plants” and “animals or yeast.” The bar height represents the number of BarleyNet links for each species group or their combination. The red bar represents the number of links that are 20 fold more likely than gene pairs by random (i.e., high confidence links).

We inferred co-functional links between barley genes by analyzing various types of omics data obtained from cultivated barley, three other plant species (*Arabidopsis thaliana*, *Zea mays*, and *Oryza sativa*), five animal species (human, *Mus musculus*, *Danio rerio*, *Caenorhabditis elegans*, and *Drosophila melanogaster*), and baker's yeast, *Saccharomyces cerevisiae*. Using our network evaluation scheme based on Bayesian statistics (see *Materials and Methods*), we selected networks with at least 2,000 inferred links more likely than those by random chance (i.e., *LLS* > 0). A total of 25 co-functional networks of barley genes inferred from distinct data sources (**Supplementary Table 1**) were integrated into a single final network mapping 1,272,200 co-functional associations between 26,145 barley genes (covering ~65.8% of 39,734 high-confidence genes) (**Figure 1A**). All edge information regarding the integrated BarleyNet and each of the component co-functional networks are freely available at the “Download” tab of the BarleyNet web server (www.inetbio.org/barleynet/download.php) and github (https://github.com/netbiolab/BarleyNet/), under the terms of the Creative Commons Attribution License (https://creativecommons.org/licenses/by-sa/4.0/).

Since a considerable amount of co-functional links were derived from other species rather than barley itself, we first summarized information sources supporting BarleyNet links (**Figure 1B**) using the UpSet visualization tool (Lex et al., 2014). We roughly classified network links into three groups based on the species of origin of the inferred co-functional association: “barley,” “other plants (*A. thaliana*, *Z. mays*, or *O. sativa*),” and “animals or yeast (human, *M. musculus*, *D. rerio*, *C. elegans*, *D. melanogaster*, or *S. cerevisiae*).” We first found that the largest portion of BarleyNet information derived from co-functional association between orthologous genes in animals or yeast (579,005 links, 45.5% of all BarleyNet links). Given that many proteins are highly conserved between unicellular eukaryote yeast and multicellular eukaryote plant species, and much information is available from yeast interactomes, the large observed contribution of yeast-derived information to BarleyNet was expected. In addition, we previously observed a large contribution of animal-derived information during the construction of co-functional gene networks for other plant species (Lee et al., 2010; Lee et al., 2019). Thus, we confirmed the usefulness of information derived from non-plant species in the reconstruction of a co-functional network of plant genes. Next, we observed a similar amount of co-functional links between barley genes was derived from the contribution of a group of other plant species. BarleyNet links derived from barley have a larger portion of links with high confidence (20-fold more likely than random gene pairs) than those derived from other plants (~26.5% compared with ~10%). This suggests that omics data generated from barley made critical contributions in improving the accuracy of BarleyNet. Finally, we noticed that only a small portion of BarleyNet links were supported by multiple species, although the majority of them are high confidence links (30–50% of links supported by two species groups and ~83.5% of links supported by all groups). Altogether, the contribution of different species groups to BarleyNet demonstrated the advantages of integrating omics data derived from various organisms in the construction of system-wide models with high completeness and accuracy.

### Barleynet Is Highly Predictive for Biological Processes in Barley

**Figure 2:**
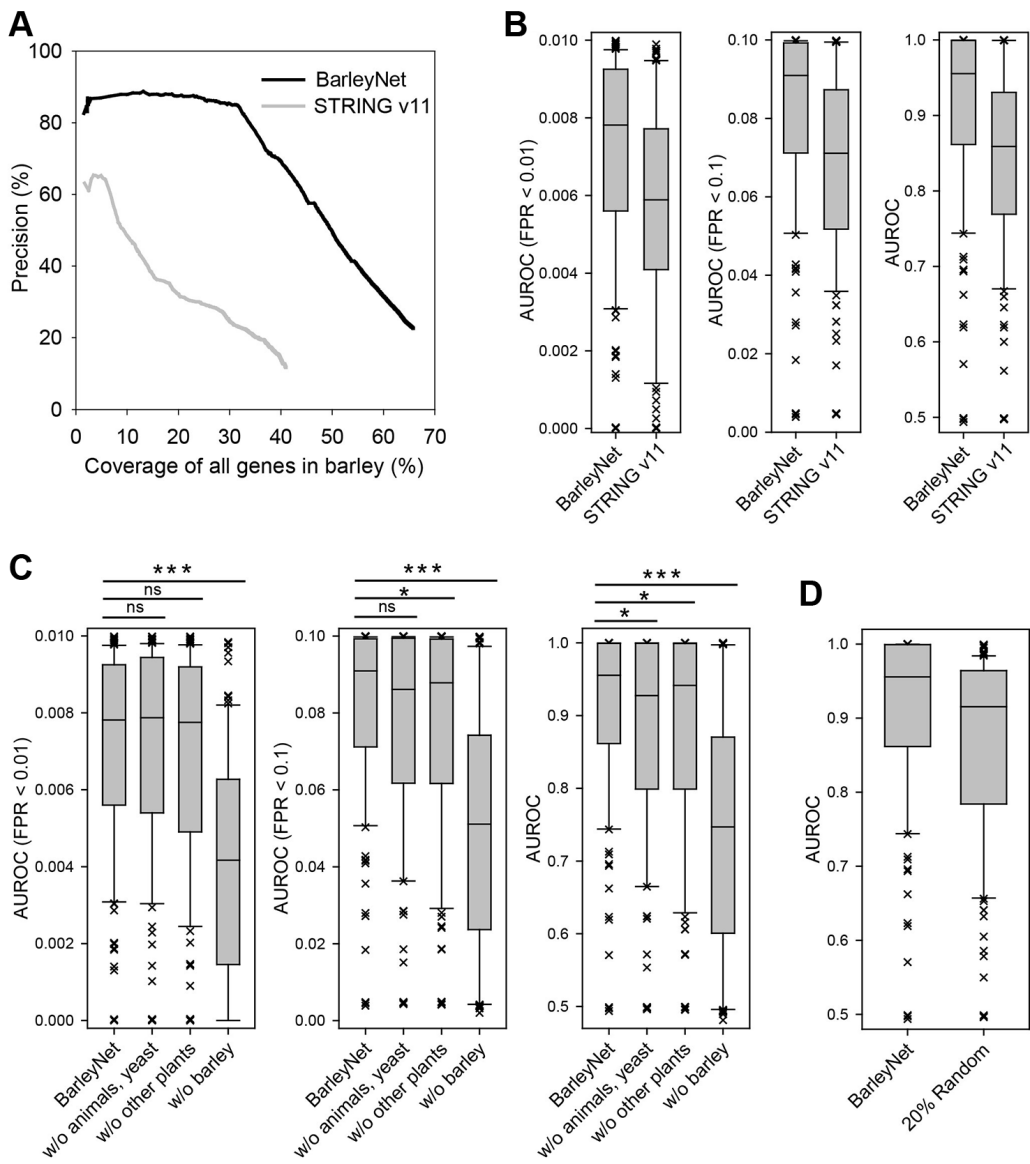
Assessment of BarleyNet and a network of barley genes by STRING database. **(A)** The quality of the networks was evaluated based on precision for gene pairs that have the same GOBP terms by agriGO annotations and coverage of all barley genes. BarleyNet showed substantially higher precision than the network of barley genes by the STRING v11 database considering the entire range of coverage. **(B)** Comparison of area under receiver operating characteristic curve (AUROC) of 122 pathway gene sets derived from Plant Reactome database. Box-and-whisker plots represent 10%, 25%, median, 75%, and 90% of 122 AUROC scores. The same AUROC analyses were conducted until 1%, 10%, and 100% of false positive rate (FPR) were reached. BarleyNet showed a significantly higher prediction power than the STRING database barley gene network in all FPR ranges (*P* < 0.001, Wilcoxon signed rank test). **(C)** AUROC analyses were conducted as for **(B)** with BarleyNet and the following “dropout” networks by excluding links from animals and yeast (w/o animals, yeast), by excluding links from *Arabidopsis*, rice, and maize (w/o other plants), and by excluding links from barley (w/o barley). ns, not significant; *, P < 0.05; ***, P < 0.001 by Wilcoxon signed rank test. **(D)** AUROC analyses were conducted as for **(B)** with 100 networks in which 20% of BarleyNet links were randomized. Average AUROC scores for the 122 pathways gene sets across 100 networks are represented in the Box-and-whisker plot for randomized networks.

We evaluated the overall quality and predictive power of BarleyNet. First, we assessed its accuracy against an existing barley gene network. To avoid circularity in network evaluation, we compiled a test dataset of gene pairs from the agriGO v2.0 database (Tian et al., 2017) which was not used for training the co-functional network of barley genes. The agriGO database provides gene ontology (GO) annotations for many agricultural animal and plant species, including barley. We found that gene pairs for the same GO biological process (GOBP) term comprised only 1.72% of gene pairs used for training BarleyNet, which indicates independence from the dataset used for network evaluation. The evaluation could be biased by gene pairs for GOBP terms that annotate a very large number of genes, so we ignored GOBP terms that annotated more than 1,000 genes during network evaluation. Subsequently, we compared BarleyNet and a barley network available at the STRING v11 database (Szklarczyk et al., 2019) regarding network accuracy (precision of gene pairs for the same GOBP terms) and coverage of all high confidence genes in barley (**Figure 2A**). We found that BarleyNet is substantially more accurate than the STRING database network of barley genes for any genome coverage. For example, in networks that cover 30% of the barley genome, the accuracy of BarleyNet is ~85.2% whereas that of the STRING database barley gene network is ~24.5%. Although the latter contains ~2.6 million links, it covers only 41% of all 39,734 high-confidence genes in barley, whereas the former covers ~65.8% of them. From these results, we concluded that BarleyNet is substantially more comprehensive and accurate than the STRING database network of barley genes.

Next, we evaluated the network-based gene prioritization for biological processes in barley. In an accurate and comprehensive co-functional network, the genes involved in same biological processes or pathways are highly likely to be connected by the network. If we prioritize genes for a particular pathway by network connections to the known genes of the pathway, all of the known pathway genes will be ranked generally higher than the others. Then, we may assess the network-based gene prioritization by receiver operating characteristic (ROC) analysis for the pathway genes, which can also be summarized as the area under the ROC curve (AUROC). We computed AUROC scores not only for entire ranks of predictions but also for early retrieved candidates, because only the top several hundred candidate genes are generally considered for the follow-up functional analysis in real practice. We thus computed AUROC until reaching false positive rates (FPRs) of 1% and 10%, in addition to AUROC for all predictions. We compared BarleyNet and the STRING database network of barley genes in the prediction of pathways annotated by the Plant Reactome database, ver. 59 (Gupta et al., 2016; Naithani et al., 2017), which was not used for training either BarleyNet or the STRING database network. We computed the AUROCs for 122 Plant Reactome pathways that annotate at least 10 barley genes and found that BarleyNet is significantly more predictive than the STRING database network for pathways with both early retrieved predictions and entire ranks of predictions (*P* < 0.001 by the Wilcoxon signed rank test for all comparisons, **Figure 2B**). From these results, we concluded that BarleyNet is substantially more predictive for various biological processes in barley than the existing STRING database gene network.

Since BarleyNet includes a large number of co-functional links between barley genes inferred from other species, we evaluated the contribution of network information originating from different species. For the analysis, we generated “dropout” networks that excluded the co-functional links derived from barley, plant species other than barley (*Arabidopsis*, rice, or maize), or animals and yeast (**Figure 2C**). We observed large decreases in the AUROCs for all range of FPRs by excluding links derived from barley. Notably, we observed significant decreases in the overall AUROC by excluding links inferred from other species, but not in the AUROCs for early-retrieved candidates (for FPR < 0.01 or 0.1). These results suggest that co-functional links transferred from other species by orthology contribute to the functional prediction, but not as much as those inferred from species-specific omics data sources.

We also tested robustness of BarleyNet-based functional prediction by evaluating networks with some degree of noise in network information. For the analysis, we generated 100 networks in which 20% of BarleyNet links were randomized while maintaining characteristics of network topology. Although, we observed significant decrease in AUROC with 20% of noise in network information, they were still higher than those by STRING database network (**Figure 2D**). This result suggests that BarleyNet-based functional prediction is relatively robust to some degree of noise in network information.

### Gene Prioritization for Complex Traits Using Barleynet

**Figure 3:**
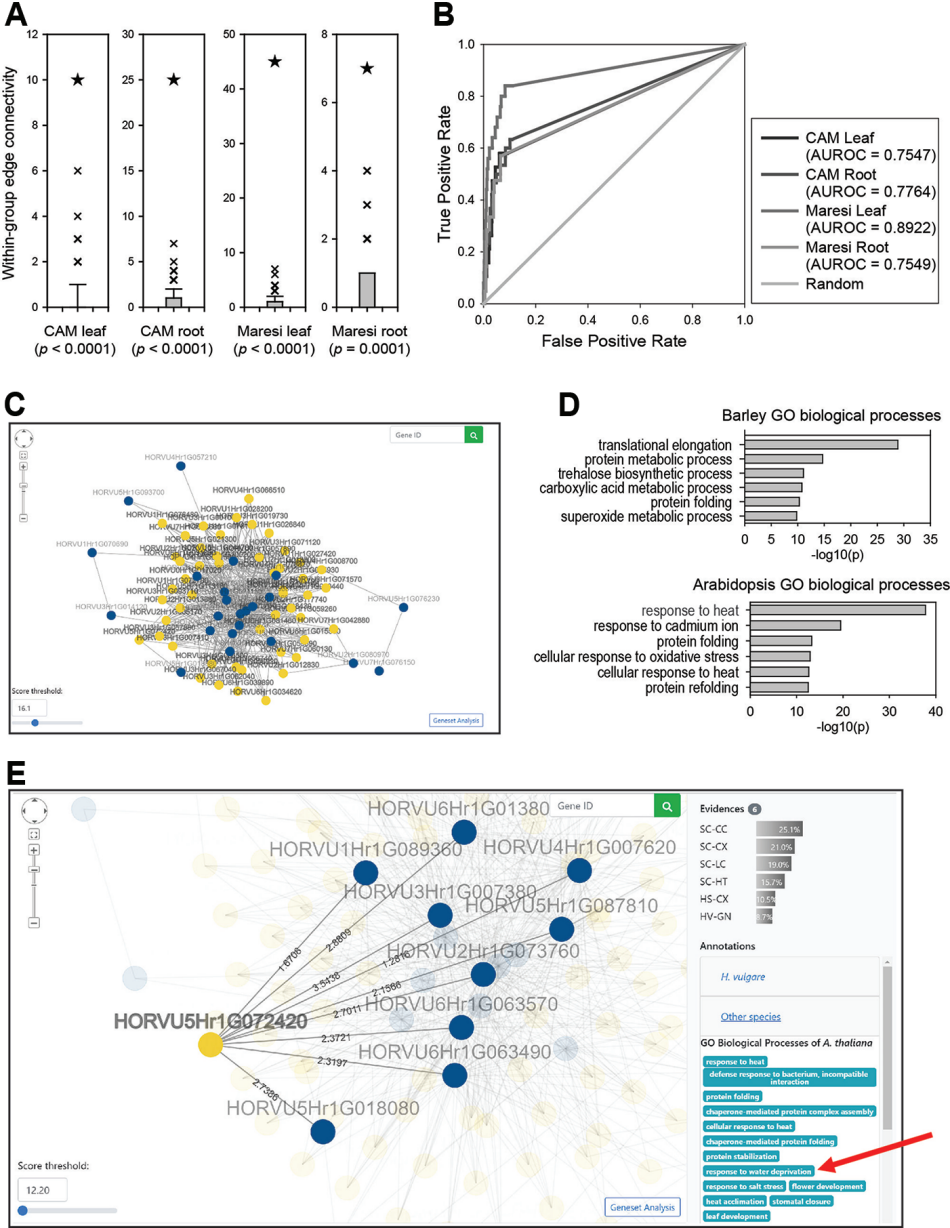
Predictions for drought response genes using BarleyNet. **(A)** Within-group edge connectivity was computed for drought response genes identified from leaves and roots of two cultivars, Maresi and Cam/B1/CI (referred to as CAM), and 1,000 random gene sets of the same size. Asterisks indicate the within-group edge count of each trait-associated gene set in BarleyNet. Within-group edge counts for drought response genes by BarleyNet were significantly higher than those by random gene sets (*P* < 0.001 by a binomial test). **(B)** AUROC analysis for the same drought response genes. **(C)** Screenshot of network viewer, which visualizes a network of drought response genes identified from differentially accumulated proteins in CAM roots (guide genes; blue nodes) and their 50 closest neighbors (candidate genes; yellow nodes) in BarleyNet. The number of neighbors in the network can be controlled by selecting a score threshold at the bottom left area. Clicking the button at the right bottom area allows gene set enrichment analysis for the selected neighbors. **(D)** Enriched GOBP terms among the 50 closest neighbors to the drought response genes, based on barley (upper plot) and *Arabidopsis* GOBP annotations (lower plot). **(E)** Screenshot of the network viewer highlighting a selected candidate gene (yellow node), HORVU5Hr1G072420. The viewer also highlights its connected user-input guide genes (i.e., drought response genes; blue nodes) and edges with their log likelihood scores. The right-side panel shows related information such as data sources that support the prediction of HORVU5Hr1G072420 as a candidate gene (Evidences) with relative contributions (% of total prediction score), as well as GOBP annotations for the candidate gene. Notably, the selected candidate gene HORVU5Hr1G072420 was annotated for “response to water deprivation” in *Arabidopsis* GOBP annotations (marked by a red arrow).

The majority of omics studies on crop species aim to identify genetic components underlying economically important and complex traits such as environmental stress responses. Through the above presented benchmarking with GOBP and Plant Reactome database, BarleyNet proved to be highly predictive for pathways, but not yet for complex traits. Most human diseases are complex traits and a large portion of human disease genes were shown to be strongly associated with specific pathways (Li and Agarwal, 2009). We thus expected that genes for complex plant traits should be associated with specific pathways, and given that BarleyNet is highly predictive for pathways, it might also be predictive for complex traits. If a network is predictive for a complex trait, the genes involved in this trait might be more connected to one another than to other genes. We thus evaluated BarleyNet in the prediction of complex traits based on the connectivity within a group of genes involved in the same traits. For this, we compiled genes for complex traits from drought-induced proteomic profiles of barley (Chmielewska et al., 2016). This study identified differentially accumulated proteins in the leaves and roots of two barley cultivars, Maresi and Cam/B1/CI (referred to as CAM), after 10 days of drought. We observed a significantly higher connectivity within a group of genes than in random gene sets of the same size in both organs of both cultivars (**Figure 3A**), which indicated that BarleyNet is significantly more predictive of drought response than random chance. The predictive power of BarleyNet for drought response was confirmed by high AUROC scores for the same groups of drought response genes (**Figure 3B**).

Considering the obtained results, we hypothesized that we might prioritize additional candidate genes for drought response through their connections to experimentally identified genes. This approach is basically a network-based search for novel candidate genes for a complex trait using previously identified genes as guides. Candidate genes were then ranked by sum of edge weight scores to the guide genes, which reflects their functional closeness. We implemented this network algorithm as a pathway-centric search method in the BarleyNet server. This server application also provides a network viewer, which visualizes a network of user-input guide genes and their closely connected neighbors. For example, **Figure 3C** shows a network of drought response genes identified from differentially accumulated proteins in CAM roots and their 50 closest neighbors. The neighbors of guide genes could be novel candidates involved in drought response in barley. Although providing a proxy for future functional studies, these candidate genes from network-based prediction should be taken with some careful consideration. The gene set analysis function of the pathway-centric search enables users to test whether these new candidates are enriched for relevant GOBP annotations. Since GOBP annotations for barley genes are still very sparse, we also employed annotations for orthologous proteins in three relatively well annotated plant species: *Arabidopsis*, rice, and maize. We found that GOBP annotations by orthology are useful in the interpretation of novel candidate genes. For example, we could not find any GOBP terms closely related to drought response among the top five enriched barley GOBP annotations. However, we found “response to heat” and “cellular response to heat,” which are closely related to drought response, among the top five enriched *Arabidopsis* GOBP annotations (**Figure 3D**). Through the BarleyNet server, users can run gene set enrichment analyses for GOBP terms of all four plant species simultaneously.

A pathway-centric search provides additional information such as the list of user-input guide genes, within-group connectivity tests and AUROC analysis results for the guide gene set, as well as the list of top 100 candidate genes. By selecting a specific candidate gene, users can obtain detailed information including its connected guide genes, edge scores, data sources that support the prediction and their relative contribution, and GOBP annotations (**Figure 3E**). For example, HORVU5Hr1G072420 was a candidate drought response gene ranked 13^th^. The network viewer informed that six distinct data sources supported the prediction, of which yeast co-citation (SC-CC) data contributed the most (25.1% of the total prediction score). Codes for all distinct data sources are listed in **Supplementary Table 1**. Notably, the candidate genes were annotated as “response to water deprivation” in *Arabidopsis* GOBP annotation but not in barley, which demonstrates the usefulness of GOBP annotations from other plant species in the interpretation of BarleyNet predictions.

### Prediction of Gene Functions Using BarleyNet

In this next step, we implemented the gene-centric search which prioritizes biological functional concepts for a gene of interest. Many proteins differentially accumulated in barley after drought stress are not yet functionally annotated. With the gene-centric search application, we can prioritize GOBP terms for genes detected in drought conditions using GOBP terms that annotate their network neighbors through information propagation. Information can be propagated to both direct and indirect neighbors in the network, and we only used the propagation to direct neighbors. We prioritized GOBP terms based on the sum of edge weight scores (log likelihood scores) to the neighbors annotated by the GOBP terms.

**Figure 4:**
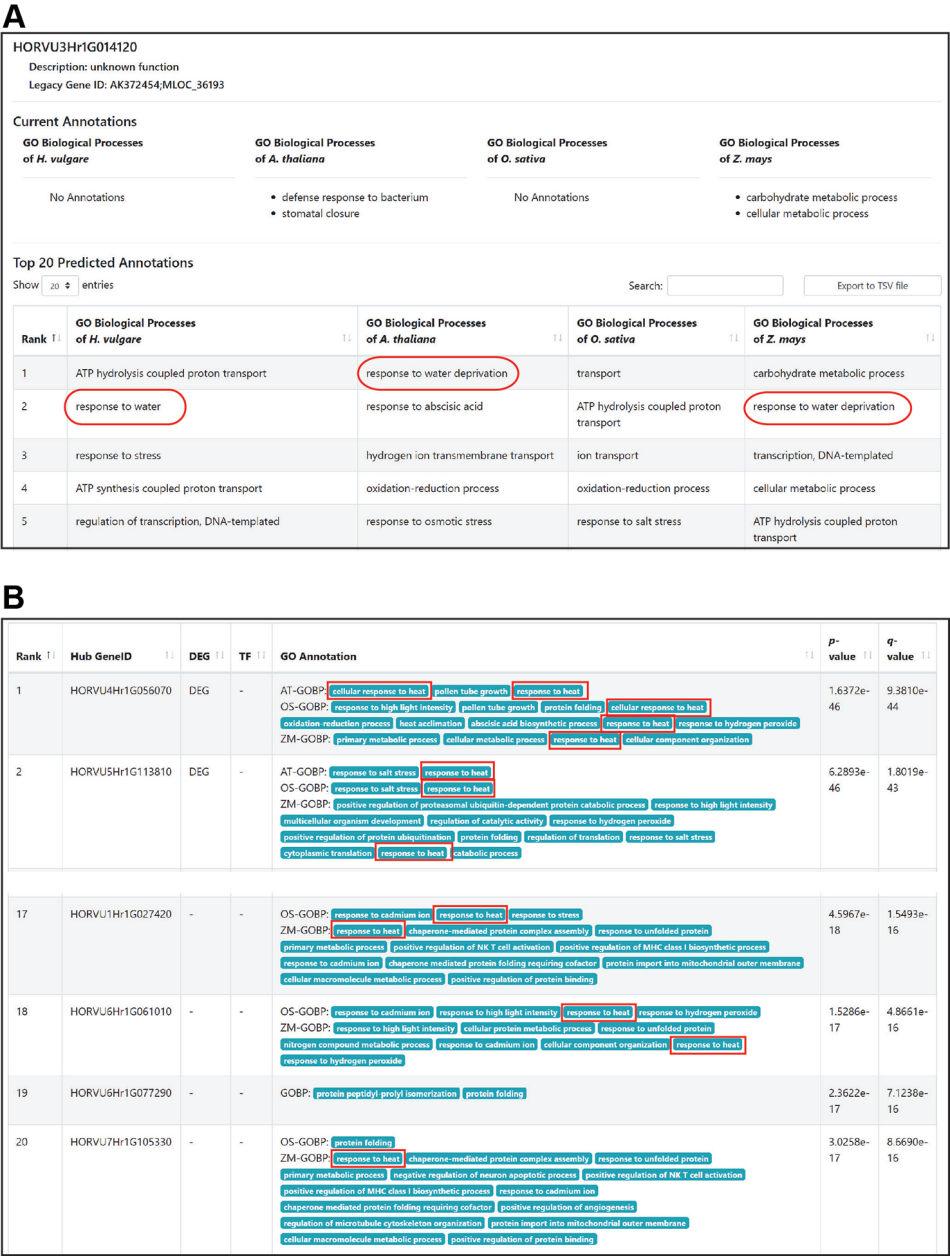
Example results from gene-centric search and context-centric search analyses using BarleyNet. **(A)** Screenshot of BarleyNet gene-centric search results with gene HORVU3Hr1G014120, which was not annotated by barley GOBP terms. GOBP terms for drought response, “response to water” and “response to water deprivation,” are marked by red circles. **(B)** Screenshots of BarleyNet context-centric search results with 625 upregulated differentially expressed genes upon heat stress in the roots of barley cultivar Rolap. The predicted genes between rank 2 and 16 were omitted. GOBP terms for heat stress response are marked by red circles.

**Figure 4A** shows a screenshot of gene-centric search results for HORVU3Hr1G014120, which was differentially accumulated in CAM roots but had no GOBP annotation yet. Gene-centric search predicted “response to water” or “response to water deprivation” genes within the top five prioritized GOBP terms according to annotations for barley, *Arabidopsis*, and maize. This example clearly demonstrated that the BarleyNet gene-centric search is a useful tool in the functional interpretation of omics data in the study of complex traits of barley.

### Prediction of Stress Response Genes Using Barleynet and Gene Expression Data

Finally, we provided context-centric search: a network-based prediction algorithm that uses differentially expressed genes (DEGs) along with the barley gene network to prioritize those associated with stress responses. In general, genes that respond to biotic or abiotic stresses are detected through genome-wide transcriptome profiling in which DEGs are considered to be involved in the stress response. However, some of the DEGs might play more important roles in stress response than others. Moreover, genes that do not change their transcript levels may also be involved in stress response. As discussed earlier, genes for complex plant traits such as stress response are likely to be associated with specific pathways. Therefore, we could prioritize genes involved in stress response by the changes in expression profiles of pathways they belong to. For this analysis, we pre-defined each gene and its direct neighbors in BarleyNet as subnetworks that represent pathways. We then selected subnetworks of “hub genes” that had at least 100 neighbors. The algorithm then computed the significance of overlap between user-submitted DEGs associated with a biological context such as stress conditions and the neighbors of each hub gene using Fisher's exact test. If the overlap between gene sets turned out to be significant, the hub gene was considered a “context-associated hub” highly likely to be involved in the biological context. The prioritized context-associated genes could be either DEGs or not.

In order to demonstrate the utility of the context-centric search application, we compiled 625 upregulated DEGs upon heat stress in barley cultivar Rolap root (Pacak et al., 2016). We manually evaluated novel candidate genes predicted by the context-centric search using the 625 upregulated DEGs (adj. p-value ≤ 0.05 and fold change ≥ 4) as input data. We found that many top ranked predictions are also DEGs that are annotated by GOBP terms for heat responses such as “response to heat” and “cellular response to heat” (**Figure 4B**). Notably, we observed candidate genes that are not DEGs but are annotated as heat response genes (see candidate genes ranked 17^th^, 18^th^, and 20^th^). These results clearly demonstrated that the network-based prediction along with functional genomics data facilitates the discovery of novel candidate stress response genes that could not be identified by expression profiles alone.

Because context-centric search uses network algorithm different from that of pathway-centric search, they are expected to provide different candidate genes. To investigate to what extent candidate genes vary by alternative network algorithms, we compared predictions by pathway-centric and context-centric searches for the same input genes, 30 drought response genes from differentially accumulated proteins in CAM roots. We found that 24 genes overlap between top 50 predictions from the two different network searches (48% overlap). Nevertheless, a functionally relevant GOBP term, “response to heat,” was found to be enriched for both of the top 50 predictions, which indicates that both network-based methods can provide highly probable candidate genes. These results also suggest that users may use the alternative network-based methods complementarily to obtain more confident candidate genes for the follow-up functional analysis.

## Data Availability Statement

The datasets generated for this study are freely available from https://github.com/netbiolab/BarleyNet/ and https://www.inetbio.org/barleynet.

## Author Contributions

SL, TL, and IL conceived the original research. SL and TL performed data analysis, constructed the network model and conducted network analysis. SY developed the web server. IL supervised the project. SL and IL wrote the manuscript with contributions from all authors. IL agrees to serve as the author responsible for contact and ensures communication.

## Conflict of Interest

The authors declare that the research was conducted in the absence of any commercial or financial relationships that could be construed as a potential conflict of interest.
