# Supplementary material for: BarleyNet: A Network-Based Functional Omics Analysis Server for Cultivated Barley, Hordeum vulgare L
Source: Front Plant Sci. 2020 Feb 18;11:98. doi: 10.3389/fpls.2020.00098 (PMC7040090; doi:10.3389/fpls.2020.00098)
Supplement: Supplementary file 1 [file Table_1.docx]

**Supplementary Table 1**. List of the 25 component networks of BarleyNet

| Network | Description | No. of Links |
| --- | --- | --- |
| HV-CX | Inferred links from co-expression patterns of two barley genes  (based on high-throughput gene expression data) | 145,000 |
| HV-DP | Inferred links from co-occurrence patterns of protein domains between barley coding genes | 40,000 |
| HV-GN | Inferred links from the similarity of genomic contexts of bacterial orthologs between barley genes | 110,000 |
| HV-PG | Inferred links from the similarity of phylogenetic profiles between barley genes | 129,000 |
| AT-CC | Associalogs from co-citation patterns between *A. thaliana* genes | 85,000 |
| AT-CX | Associalogs from co-expression patterns between *A. thaliana* genes  (based on high-throughput gene expression data) | 324,000 |
| AT-HT | Associalogs from high-throughput protein-protein interactions between *A. thaliana* genes | 2,083 |
| AT-LC | Associalogs from literature-curated protein-protein interactions between *A. thaliana* genes | 3,984 |
| CE-CX | Associalogs from the co-expression pattern between *C. elegans* genes  (based on high-throughput gene expression data) | 74,000 |
| DM-CX | Associalogs from co-expression patterns between *D. melanogaster* genes  (based on high-throughput gene expression data) | 110,000 |
| DM-HT | Associalogs from high-throughput protein-protein interactions between *D. melanogaster* genes | 7,000 |
| DM-LC | Associalogs from literature-curated protein-protein interactions between *D. melanogaster* genes | 2,501 |
| DR-CX | Associalogs from co-expression patterns between *D. rerio* genes  (based on high-throughput gene expression data) | 100,000 |
| HS-CX | Associalogs from co-expression patterns between *H. sapiens* genes  (based on high-throughput gene expression data) | 64,000 |
| HS-HT | Associalogs from high-throughput protein-protein interactions between *H. sapiens* genes | 64,000 |
| HS-LC | Associalogs from literature-curated protein-protein interactions between *H. sapiens* genes | 78,000 |
| MM-CX | Associalogs from co-expression patterns between *M. musculus* genes  (based on high-throughput gene expression data) | 72,000 |
| MM-LC | Associalogs from literature-curated protein-protein interactions between *M. musculus* genes | 14,000 |
| OS-CX | Associalogs from co-expression patterns between *O. sativa* genes  (based on high-throughput gene expression data) | 303,000 |
| SC-CC | Associalogs from co-citation patterns between *S. cerevisiae* genes | 202,000 |
| SC-CX | Associalogs from co-expression patterns between *S. cerevisiae* genes  (based on high-throughput gene expression data) | 98,000 |
| SC-GT | Associalogs from genetic interaction patterns between *S. cerevisiae* genes | 86,000 |
| SC-HT | Associalogs from high-throughput protein-protein interactions between *S. cerevisiae* genes | 57,000 |
| SC-LC | Associalogs from literature-curated protein-protein interactions between *S. cerevisiae* genes | 90,000 |
| ZM-CX | Associalogs from co-expression patterns between *Z. mays* genes  (based on high-throughput gene expression data) | 53,000 |
