# Supplementary material for: BarleyNet: A Network-Based Functional Omics Analysis Server for Cultivated Barley, Hordeum vulgare L
Source: Front Plant Sci. 2020 Feb 18;11:98. doi: 10.3389/fpls.2020.00098 (PMC7040090; doi:10.3389/fpls.2020.00098)
Supplement: Supplementary file 2 [file Table_2.docx]

**Supplementary Table 2**. List of 28 gene expression datasets used to construct co-expression networks

| Accession ID | Dataset description |  | No. of Links |
| --- | --- | --- | --- |
| GSE9365 | Expression data from barley maturing and germinating grains | Microarray (Affymetrix) | 68,000 |
| GSE11200 | Expression data from malting barley seeds | Microarray (Affymetrix) | 72,000 |
| GSE14930 | Comparison of wild-type and cell death mutant of barley plants containing Mla6 powdery mildew resistance gene | Microarray (Affymetrix) | 240,000 |
| GSE15970 | Differentially Expressed Genes between Drought-tolerant and Drought-sensitive Barley Genotypes | Microarray (Affymetrix) | 21,000 |
| GSE17669 | Gene expression in the barley spike during drought stress | Microarray (Affymetrix) | 40,000 |
| GSE20279 | *Blumeria graminis* fsp hordei effects on translation initiation in compatible and incompatible barley | Microarray (Affymetrix) | 230,000 |
| GSE20416 | Genetic regulation of gene expression of barley in response to stem rust (Pgt isolate TTKS) | Microarray (Affymetrix) | 314,000 |
| GSE27821 | Short and long term cold responses in a winter barley | Microarray (Affymetrix) | 126,000 |
| GSE33392 | Transcription profiling of barley plants containing variants of Mla1 and Mla6 powdery mildew resistance genes | Microarray (Affymetrix) | 226,000 |
| GSE33393 | Genotype-dependent gene expression in barley | Microarray (Affymetrix) | 184,000 |
| GSE33396 | Mla-specified Transcriptional Responses in Barley-Powdery Mildew Interactions | Microarray (Affymetrix) | 266,000 |
| GSE33401 | Transcriptome analysis of wild barley (*H. vulgare* ssp spontaneum) after pathogen inoculation | Microarray (Affymetrix) | 108,000 |
| GSE33407 | Barley cv Morex inoculated with *Fusarium graminearum* and water as mock control | Microarray (Affymetrix) | 50,000 |
| GSE43704 | Comparison of isogenic lines differing in vernalization requirement | Microarray (Affymetrix) | 104,000 |
| GSE43906 | Transcript profiling of local and adjacent leaf responses in barley following inoculation with *Pseudomonas syringae* | Microarray (Affymetrix) | 24,000 |
| GSE56437 | Transcriptome characterization of genotype-dependent effects of CaCl2 treatment on drought adaptation in barley (*Hordeum vulgare* L.). | Microarray (Affymetrix) | 112,000 |
| GSE61644 | Transcript profiling of Bln1 silenced plants (BSMV-VIGS) relative to empty vector and buffer treated controls in barley-powdery mildew interactions | Microarray (Affymetrix) | 132,000 |
| GSE64228 | Expression data of leaves from transgenic barley expressing wheat Lr34 gene | Microarray (Affymetrix) | 60,000 |
| GSE68963 | Genetic regulation of barley gene expression in response to the powdery mildew fungus, *Blumeria graminis* f. sp. hordei (Bgh) | Microarray (Affymetrix) | 36,000 |
| E-TABM-82 | Transcription profiling of wild type and mutants of Sultan V barley plants | Microarray (Affymetrix) | 77,000 |
| E-TABM-142 | Transcription profiling of wild type and loss-of-function mutants of Mla1 and Mla6 powdery mildew resistant allele barley plants | Microarray (Affymetrix) | 164,000 |
| GSE83676 | Barley transcriptional responses to specialist and generalist spider mite herbivores | RNA-seq (HiSeq 2500) | 36,000 |
| GSE101304 | Barley CI 16151 and fast-neutron-derived, immune-compromised mutants infected with the powdery mildew fungus (*Blumeria graminis* f. sp. hordei; isolate 5874) | RNA-seq (HiSeq 2500) | 310,000 |
| ERP001600 | RNA-seq of coding RNA of eight barley tissues from different developmental stages | RNA-Seq (HiSeq 2000) | 150,000 |
| ERP009781 | Expression profiling of developing barley main shoot apices and leaves during the vegetative phase and early inflorescence development in response to the photoperiod and natural allelic variation at Ppd-H1 | RNA-Seq (HiSeq 2000) | 109,000 |
| ERP014029 | RNAseq transcriptome assembly and expression profiling of a drought-control experiment with barley cultivar Scarlett and Spanish landrace SBCC073. | RNA-Seq (HiSeq 2000) | 103,000 |
| ERP015182 | A study of transcriptome response to salt treatment across three zones of the root in a barley landrace and malting cultivar | RNA-Seq (HiSeq 2000) | 130,000 |
| ERP015986 | RNA-Seq of 16 developmental stages of barley (Morex cultivar) | RNA-Seq (HiSeq 2000) | 102,000 |
